# Management of osteonecrosis of the humeral head in the pediatric population: A systematic review

**DOI:** 10.1177/18632521241254708

**Published:** 2024-05-19

**Authors:** Vineet M Desai, Akbar N Syed, Morgan Batley, Lawrence Wells, Brendan A Williams

**Affiliations:** 1 Department of Orthopaedics, The Children’s Hospital of Philadelphia, Philadelphia, PA, USA; 2 Harvard Medical School, Boston, MA, USA

**Keywords:** Humeral head, avascular necrosis, osteonecrosis, pediatric

## Abstract

**Purpose:**

Humeral head osteonecrosis in the pediatric patients most often occurs in patients with underlying hemoglobinopathies, exposure to chronic corticosteroids, or after trauma. The purpose of this study was to perform a systematic review evaluating the prevalence, clinical characteristics, and management of humeral head osteonecrosis in the pediatric population.

**Methods:**

PubMed, Ovid MEDLINE, and Scopus were screened with the terms “osteonecrosis,” “avascular necrosis,” “pediatric,” and “proximal humerus” on January 10, 2024. A total of 218 studies were screened, and 74 studies were evaluated for eligibility. Studies that reported on the prevalence and/or management of pediatric humeral head osteonecrosis were included. The systematic review was conducted according to the Preferred Reporting Items for Systematic Review and Meta-Analyses guidelines.

**Results:**

Twelve studies met inclusion criteria: four retrospective case series, three prospective case series, one retrospective cohort study, one retrospective case-control study, and three case reports. A majority of the studies (67%) discussed chemotherapy-induced osteonecrosis of the humeral head. A total of 77 patients (106 shoulders) with humeral head osteonecrosis were identified. The overall prevalence of osteonecrosis of the humeral head across eight studies examining at-risk populations (underlying hemoglobinopathies or undergoing chemotherapy) was 2%. Intra-articular steroid injections, physical therapy, and activity modification are effective conservative management strategies. Additionally, core decompression and hemiarthroplasty are surgical treatment options.

**Conclusions:**

The prevalence of osteonecrosis of the humeral head is low even among at-risk populations with associated medical conditions. A variety of conservative and surgical treatment options have been described, but no comparative evaluations of these modalities has been conducted.

**Level of evidence:**

IV.

## Introduction

Osteonecrosis, also known as avascular necrosis (AVN), is caused by the disruption of blood supply to the bone.^1^ While it is commonly associated with the femoral head, osteonecrosis can also often occur at the humeral head, femoral condyles, talus, and small carpal and tarsal bones.^2^ Osteonecrosis in pediatric and young adult populations can present, either focally or systemically, in the context of a variety of medical and iatrogenic abnormalities: trauma, hemoglobinopathies such as sickle-cell disease (SCD) or thalassemia, long-term corticosteroid usage, and rheumatologic conditions.^2–4^

Although osteonecrosis of the proximal humerus is the second most common site of osteonecrosis after the femoral head, it is poorly understood and difficult to diagnose.^5,6^ Patients with humeral head osteonecrosis are often asymptomatic or have minimal pain, and the progression of this condition is slow due to the lesser weight-bearing status of the glenohumeral joint in comparison to the lower extremity.^5,7^ Diagnosis may initially be incidental, identified on advanced imaging performed for other purposes. However, patients with humeral head osteonecrosis often have concurrent multifocal osteonecrosis, present radiographically with collapse of the humeral head and sclerosis, and present clinically with pain, limited range of motion (ROM), and functional limitations.^6,8–11^

Humeral head osteonecrosis is typically treated initially with conservative management such as physical therapy, anti-inflammatory medications, and activity modification.^12^ When conservative management fails or osteonecrosis has advanced substantially toward articular collapse, patients undergo surgical management options including core decompression (CD), resurfacing, hemiarthroplasty, or total shoulder arthroplasty.^13,14^ Franceschi et al.^5^ conducted a systematic review of surgical management of osteonecrosis of the humeral head in the adult population and found that whereas CD is effective for low-grade osteonecrosis, arthroplasty should be considered for high-grade osteonecrosis. However, literature remains limited regarding the characteristics and management of humeral head AVN in younger populations.^5^ The purpose of this study was to perform a systematic review to improve our understanding of the existing evidence regarding the prevalence and characteristics of proximal humeral AVN in young patients, the treatment modalities utilized, and the outcomes of these treatments in this population.

## Materials and methods

### Search strategy

**Figure 1.**
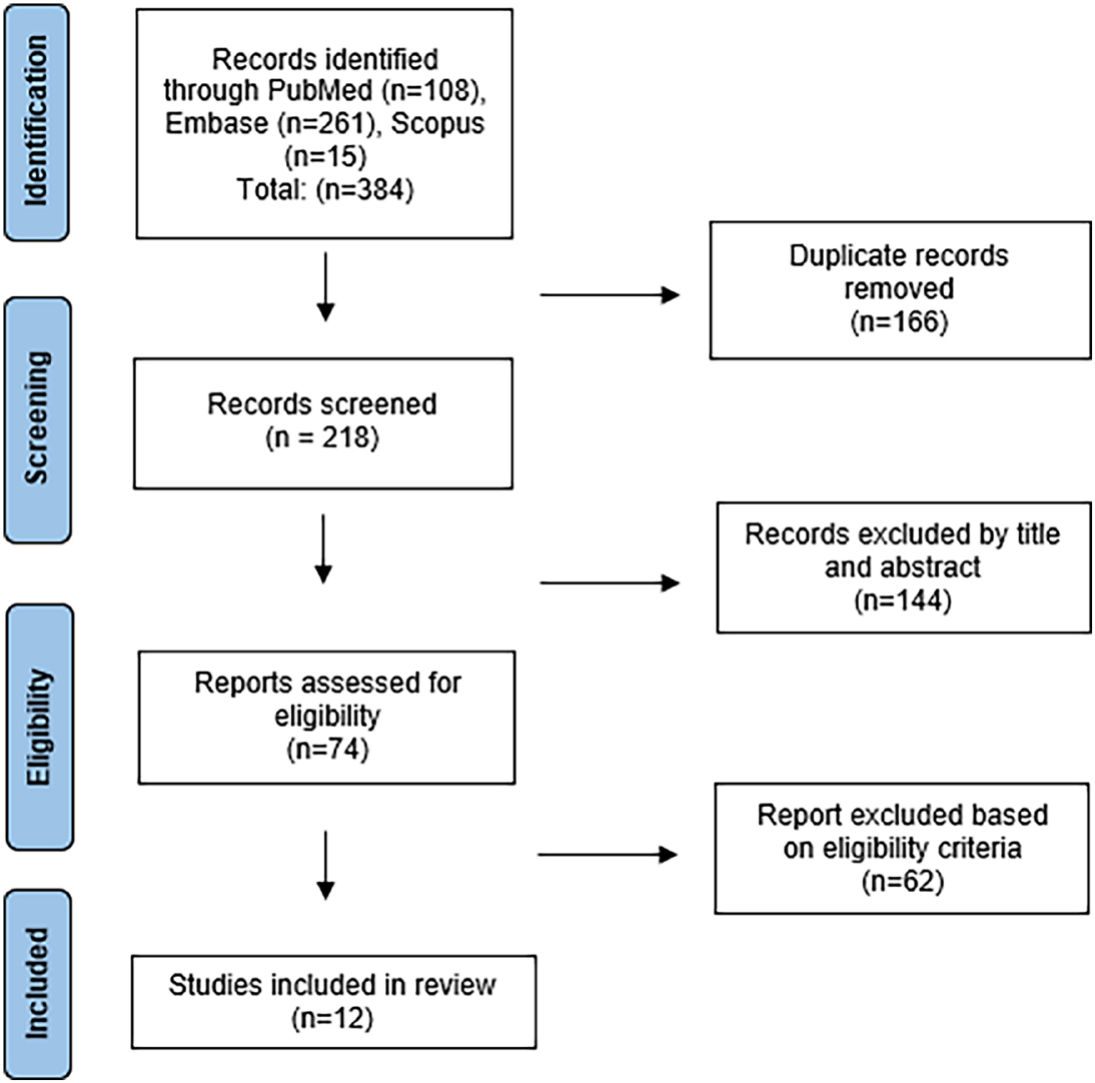
Study selection flowchart using PRISMA guidelines.

We performed a systematic review identifying published literature examining the prevalence and/or management of osteonecrosis of the humeral head in the pediatric population, following the Preferred Reporting Items for Systematic Reviews and Meta-Analyses (PRISMA) and Cochrane collaboration guidelines (Figure 1; Supplemental material Appendix 1). We searched the PubMed, OVID Embase, and Scopus databases with the following search string: (avn OR osteonecrosis [MeSH Terms] OR “avascular necrosis” OR “aseptic necrosis”) AND (Child OR Pediatric) AND (“proximal humerus” OR “humeral head” OR shoulder). The search was executed on January 10, 2024.

### Eligibility criteria

Studies were excluded if they did not have a full-text article available or were presented in a non-English language. In assessing full-text articles, studies were excluded if they either did not report on the prevalence and/or management of humeral head osteonecrosis, or if they did not have extractable data specifically on a majority pediatric population (age <21). Additionally, review papers, editorials, and technique papers were excluded.

### Study selection

Databases were searched from inception to our search date. After the search was performed, duplicate studies were removed, and we screened the title/abstract of studies based on relevance to osteonecrosis of the humeral head. The full texts for the remaining studies were then retrieved and evaluated based on eligibility criteria. The bibliography of each published study was reviewed for additional relevant articles that may have been potentially missed. Two authors analyzed and reviewed each article individually (A.N.S. and M.B.), and in the event of disagreement for inclusion, the senior author (B.A.W.) acted as the tie breaker.

### Data collection and data analysis

Each study was classified as prospective cohort, retrospective cohort, case series, or case report. Level of evidence, total number of patients in cohort, total number of patients with osteonecrosis, total number of pediatric patients with osteonecrosis of the humeral head, age, and length of follow-up were extracted from each study, as applicable. Clinical characteristics (etiology of osteonecrosis, imaging, grade of osteonecrosis, and symptoms) and management characteristics (conservative versus operative management, reported interventions, and outcome of intervention) were collected as well. Using a target population including patients with hemoglobinopathy or those undergoing chemotherapy, prevalence was calculated as the total number of patients/shoulders with osteonecrosis of the humeral head divided by the total number of patients/shoulders at risk. Case reports were not included in either the total identified humeral heads with osteonecrosis or the prevalence calculation. Data from each study were sorted into one or more of three categories related to pediatric osteonecrosis of the humeral head: prevalence, conservative management, and surgical management. The data was synthesized and visually displayed using Microsoft Excel (2021 version, Microsoft Corporation, Redmond, WA, USA).

### Risk of bias assessment

Two independent reviewers (V.M.D. and A.N.S.) assessed the risk of bias within each study. Bias was analyzed using the Cochrane Risk of Bias Assessment Tool: for nonrandomized studies of interventions for cohort and case control studies as well as the Joanna Briggs Institute (JBI) critical appraisal tool for case series.^15,16^ If there was discordance between the two reviewers, the senior author (B.A.W.) served as the tie breaker.

### Institutional review board (IRB) approval

This systematic review is exempt from IRB approval.

## Results

### Study selection and characteristics

A total of 218 studies were identified through the electronic search. After initial screening and eligibility review, 12 studies remained eligible for inclusion in this systematic review. These studies included three prospective case series,^17–19^ four retrospective case series,^20–23^ one retrospective case-control study,^24^ one retrospective cohort study,^25^ and three case reports.^26–28^ Eight studies reported exclusively on the pediatric population,^17,18,22–24,26–28^ while four studies had mixed cohorts.^19–21,25^ Eight studies discussed osteonecrosis of the humeral head as a side effect of chemotherapy used to treat underlying Acute Lymphoblastic Leukemia/Lymphoma, Hodgkin’s Lymphoma, or Non-Hodgkin’s Lymphoma.^17,18,20–23,25,27^ Three studies discussed osteonecrosis of the humeral head due to underlying sickle cell anemia or other related sickle cell thalassemia.^19,24,28^ One study discussed osteonecrosis of the humeral head due to a Salter-Harris II physeal fracture.^26^ The details of each individual study are presented in Table 1.

**Table 1.** Characteristics of Evaluated Studies.

Study	Level of evidence and study type		Year	Study period	Study population	Number of patients		Median age in years (range)	Mean length of follow-up in years (range)
						Total (number of shoulders)	Humeral head osteonecrosis (no. of shoulders)		
Milner et al.*	IV	Prospective Case Series	1993	1979–1981	SCD	1019 (2038)	19 (NR)	NR (5–14)	5.6 (NR)
Inaba et al.	IV	Prospective Case Series	2020	2012–2017	ALL/L	15 (30)	8 (15)	14 (9–17)	NR
Kaste et al.	IV	Retrospective Case Series	2019	1996–2014	ALL/NHL	1478 (2956)	33 (62)	14.2 (4.3–19)	6.4 (0–12.7)
Mesleh Shayeb et al.	III	Retrospective Case-Control	2018	1998–2014	SCD	612 (1224)	6 (8)	NR (6–18)	NR
Littooij, et al.	IV	Prospective Case Series	2017	2012–2015	HL	24 (48)	NR (2)	15.1 (10.1–17.9)	1.0 (0.48–3.6)
Heneghan et al.	III	Retrospective Cohort	2016	2004–2012	ALL	10,729 (21,458)	NR	7.04 (2.02–21.2)	NR
Kuhlen et al.	IV	Retrospective Case Series	2014	2003–2009	ALL	124 (248)	5 (8)	12.6 (2.4–19.9)	2.3 (0.1–6.2)
Miettunen et al.	IV	Retrospective Case Series	2012	2006–2008	ALL	32 (64)	5 (9)	5.4 (4.8–11.9)	NR
Riccio et al.	IV	Retrospective Case Series	2016	1982–2003	ALL	328 (656)	1 (2)	Mean: 7.2 SD: 0.1–14.3	NR
Wong et al.	IV	Case report	2022	—	SCD	1 (2)	1 (1)	12	0.2
Solarino et al.	IV	Case report	2008	—	ALL	1 (2)	1 (2)	12	5.3
Martin et al.	IV	Case report	1997	—	Salter-Harris II fracture	1 (2)	1 (1)	14	1
Total (of reported)**						3608 (5226)	77 (106)		

ALL/L: Acute Lymphoblastic Leukemia/Lymphoma; ALL: Acute Lymphoblastic Leukemia; HL: Hodgkin’s Lymphoma; NHL: Non-Hodgkin’s Lymphoma; NR: Not reported; SCD: Sickle Cell Disease.

* Only information regarding patient group 5–14 year included.

** Does not include data from case reports or Heneghan et al.

### Prevalence and clinical characteristics

Across eight studies that presented data for the number of patients with humeral head osteonecrosis within a greater at-risk population, there were 106 shoulders (77 patients) that developed osteonecrosis of the humeral head, and an overall at-risk population of 5226 shoulders (3608 patients). One study did not report the specific number of humeral heads that developed osteonecrosis.^25^ Thus, we calculated the overall prevalence of osteonecrosis of the humeral head within the at-risk pediatric population to be 2.0%. Across four studies, there were 21 reported humeral head osteonecrosis lesions among 246 osteonecrosis lesions across all anatomic sites. Thus, the prevalence of osteonecrosis in the humeral head among all potential osteonecrosis sites was 8.5%. Eight studies reported on the presence of multifocal osteonecrosis.^17,19–23,27,28^ Of the 73 patients with humeral head osteonecrosis across these eight studies, 69 (94.5%) had multifocal osteonecrosis.^17,19–23,27,28^ Pain was the most frequently reported symptom on presentation for patients with osteonecrosis of the humeral head as reported in nine studies,^17,19–21,23,24,26–28^ followed by loss of function,^20,27,28^ loss of ROM,^19,20^ and asymptomatic.^22^ Kaste et al.^20^ found that 43 humeral heads (69%) with osteonecrosis had 30% or greater epiphyseal involvement on magnetic resonance imaging (MRI), while 19 humeral heads (31%) had less than 30% epiphyseal involvement. Inaba et al.^17^ found that osteonecrosis of the humeral head was highest after Re-induction phase II of chemotherapy, where of 20 evaluable humeral heads seven had >30% epiphyseal involvement, three had <30% epiphyseal involvement, and one had metaphyseal involvement (Table 2).

**Table 2.** Clinical Characteristics of Humeral Head Osteonecrosis in Evaluated Studies.

Study	Etiology	Imaging	Total shoulders (symptomatic)	Osteonecrosis classification	Reported symptoms
Milner et al.	Sickle Cell Disease***	X-ray*	NR	NR	Pain, decreased ROM
Inaba et al.	Chemotherapy	WB-MRI*	15 (8)	Metaphyseal vs. <30% epiphyseal vs. >30% epiphyseal	Pain
Kaste et al.	Chemotherapy	X-ray; MRI**	43 (43)	>30% epiphyseal on MRI	Pain, loss of function, loss of ROM
			19 (19)	<30% epiphyseal on MRI	
Mesleh Shayeb et al.	Sickle Cell Disease	X-ray; MRI**	8 (8)	NR	Pain
Littooij et al.	Chemotherapy	MRI*	NR	NR	NR
Heneghan et al.	Chemotherapy	None	NR	NR	NR
Kuhlen et al.	Chemotherapy	X-ray; MRI**	8 (8)	NR	Pain
Miettunen et al.	Chemotherapy	WB-MRI	9 (0)	Based on involvement of the weight-bearing portion: Type A: <1/3rd, Type B: <2/3rd and Type C: >2/3rd	Asymptomatic
Riccio et al.	Chemotherapy	X-ray**	2 (2)	NR	Pain
Wong et al.	Sickle Cell Disease	NR	1 (1)	NR	Pain and loss of function
Solarino et al.	Chemotherapy	X-ray	2 (2)	NR	Pain and loss of function
Martin et al.	Post-Traumatic (Physeal fracture)	X-ray; MRI	1 (1)	NR	Pain

ADL: Activities of Daily Living; NR: Not Reported; WB-MRI: Whole Body MRI.

* All patients in the study sample were screened with this imaging modality, irrespective of symptomology.

** Only symptomatic patients were screened.

*** Includes Sickle Cell Anemia, Sickle Cell Beta-thalassemia, Sickle Cell-Hb C disease, Sickle-Cell Beta+-thalassemia as well.

### Conservative management

Six studies reported on conservative management of osteonecrosis of the humeral head. Inaba et al.^17^ prospectively reviewed the development of osteonecrosis in pediatric patients receiving chemotherapy. They found that when nine shoulders with >30% epiphyseal humeral head osteonecrosis were treated with chemotherapeutic dose reduction or cessation, three shoulders experienced osteonecrosis regression.^17^ Kaste et al.^20^ conducted a retrospective review of pediatric patients with glucocorticoid-induced osteonecrosis of the humeral head. They used the National Cancer Institute’s Common Terminology Criteria for Adverse Events (CTCAE) scoring system to show an improvement in the impact of osteonecrosis on activities of daily living (ADL) (2.61–1.76), pain (2.69–1.23), and ROM (2.15–1.69) after intra-articular steroid injection.^20^ Riccio et al.^23^ and Martin et al.^26^ evaluated the outcomes of patients with humeral head osteonecrosis who underwent conservative therapies such as activity modification and physical therapy. Riccio et al.^23^ found that activity modification and physical therapy led to good ROM, and Martin et al.^26^ found that activity modification led to resolution of pain. Wong et al.^28^ found in a case report that although an intensive pain rehabilitation program and steroid injections did not result in improvement in shoulder pain, intra-articular hyaluronic acid injections resulted in an over 50% reduction in pain. Lastly, Kuhlen et al.^21^ retrospectively reviewed patients with symptomatic humeral head osteonecrosis, and reported that conservative management of some of these patients included bisphosphonates and Iloprost in addition to anti-inflammatory medications and physical therapy (Table 3). Of the four patients across four studies that underwent conservative measures such as activity modification or physical therapy, three (75%) demonstrated improvement in pain and functional outcomes.

**Table 3.** Conservative Management of Humeral Head Osteonecrosis in Evaluated Studies.

Study	Shoulders	Reported interventions	Outcome
Inaba et al.	NR	Reduction or cessation of chemotherapy	>30% epiphyseal involvement: 3/9 shoulder regressed <30% epiphyseal involvement: 1 shoulder resolved
Kaste et al.	13	Intra-articular steroid injections	7/13 shoulders resolved; Mean CTCAE score for ROM improved from 2.15 to 1.69; Mean CTCAE score for pain improved from 2.69 to 1.23
	NR	Physical therapy; Anti-inflammatory agents	NR
Kuhlen et al.	8	Physiotherapy; Activity modification; Anti-inflammatory agents; bisphosphonates; Iloprost	NR
Riccio et al.	2	Activity modification; Physical therapy	Good ROM; residual humeral head deformity
Wong et al.	1	Physical therapy; Psychotherapy; Acupuncture; Intra-articular steroid injections; pain medication (pregabalin, meloxicam, methadone; hydrocodone as needed)	No improvement in pain
		Intra-articular hyaluronic acid injections	50% reduction in pain (6/10–0/10 at rest, 10/10–5/10 with activity); improvement in function
Martin et al.	1	Activity modification	Asymptomatic

ALL/L: Acute Lymphoblastic Leukemia/Lymphoma; ALL: Acute Lymphoblastic Leukemia; HL: Hodgkin’s Lymphoma; NHL: Non-Hodgkin’s Lymphoma; NR: Not reported; SCD: Sickle Cell Disease; CTCAE: National Cancer Institute’s Common Terminology Criteria for Adverse Events.

### Surgical management

Three studies reported on surgical management of osteonecrosis of the humeral head. Kaste et al.^20^ found that among 12 shoulders with osteonecrosis, nine experienced resolution after CD. Additionally, the mean CTCAE scores improved for pain (2.75–1.00) and impact on ADL (2.91–1.66) and slightly worsened for ROM (2.00–2.08) after undergoing CD.^20^ Mean CTCAE scores improved for impact on ADL (2.75–1.75), pain (2.87–0.85), and ROM (2.37–1.87) after undergoing resurfacing hemiarthroplasty.^20^ Inaba et al.^17^ reported on three shoulders with osteonecrosis that underwent surgical management, with two undergoing CD and one undergoing bone resurfacing. Neither of the patients who underwent CD experienced improvement in osteonecrosis.^17^ Of the 11 patients across these two studies that underwent CD, nine (81.8%) demonstrated resolution of osteonecrosis. Lastly, Heneghan found that of eight patients who underwent surgical intervention at the humerus for osteonecrosis, only one underwent total shoulder arthroplasty^25^ (Table 4).

**Table 4.** Surgical Management of Humeral Head Osteonecrosis in Evaluated Studies.

Study	Shoulders	Procedure	Outcome
Inaba et al.	3	CD: 2	No shoulders resolved
		Bone resurfacing: 1	NR
Kaste et al.	20	CD: 12	9/12 shoulders resolved; Mean CTCAE score for ROM worsened from 2.00 to 2.08; Mean CTCAE score for pain improved from 2.75 to 1.00
		Hemiarthroplasty (resurfacing): 8	Mean CTCAE score for ROM improved from 2.37 to 1.87; Mean CTCAE score for pain improved from 2.87 to 0.75
Heneghan et al.	8	NR	NR
	1	Total shoulder arthroplasty: 1	NR

CTCAE: National Cancer Institute’s Common Terminology Criteria for Adverse Events; NR: Not reported; ROM: range of motion.

### Risk of bias

Overall, the one retrospective cohort study and one retrospective case-control study had a low risk of bias (Table 5). The four retrospective and three prospective case series had good methodological quality (Table 6).

**Table 5.** Consensus ACROBAT-NRSI Judgments Between Two Reviewers by Domain of Bias of Included Cohort Studies.

Study	D1: bias due to confounding	D2: selection of participants	D3: bias in measurement of interventions	D4: bias due to departure from intended intervention	D6: bias due to missing data	D7: bias in selection of reported results	Overall risk of bias assessment
Mesleh Shayeb et al.	Low	Low	Low	Low	Low	Low	Low
Heneghan et al.	Low	Low	Low	Low	Low	Low	Low

**Table 6.** JBI Critical Appraisal Tool for Case Series Studies.

Study	Q1: Was there clear criteria for inclusion?	Q2: Was the condition measured in a standard, reliable way?	Q3: Were valid methods used for identification of the condition?	Q4: Consecutive inclusion of participants?	Q5: Complete inclusion of participants?	Q6: Clear reporting of demographics of participants?	Q7: Clear reporting of clinical information?	Q8: Outcomes or follow-up clearly reported?	Q9: Clear reporting of the presenting sites/clinics’ demographic information?	Q10: Statistical analysis appropriate?
Milner et al.	Yes	Yes	Yes	Yes	Yes	Yes	Yes	Yes	Yes	Yes
Inaba et al.	Yes	Yes	Yes	Yes	Yes	Yes	Yes	Yes	Yes	Yes
Kaste et al.	Yes	Yes	Yes	Yes	Yes	Yes	Yes	Yes	Yes	Yes
Littooij et al.	Yes	Yes	Yes	Yes	Yes	Yes	Yes	Yes	Yes	Yes
Kuhlen et al.	Yes	Yes	Yes	Yes	Yes	Yes	Yes	Yes	Yes	Yes
Miettunen et al.	Yes	Yes	Yes	Yes	Yes	Yes	Yes	Yes	Yes	Yes
Riccio et al.	Yes	Yes	Yes	Yes	Yes	Yes	Yes	Yes	Yes	Yes

## Discussion

Although rarer than in the hip, osteonecrosis of the humeral head, which often presents in patients with underlying hematologic conditions or with chronic exposure to glucocorticoids, can have significant morbidity.^29^ Literature regarding the most effective management strategies for osteonecrosis of the humeral head in the pediatric population is limited.^5^ The goal of this systematic review was to summarize published studies and current evidence on the prevalence and clinical characteristics, conservative management, and surgical management of osteonecrosis of the humeral head within the pediatric population. The overall prevalence of osteonecrosis of the humeral head across eight studies was about 2%. Furthermore, only a few studies highlighted the efficacy of nonsurgical interventions such as intra-articular injections and surgical interventions such as CD and hemiarthroplasty in improving the impact on ADLs, pain, and ROM for patients with humeral head osteonecrosis.

Few studies have published on the prevalence of humeral head osteonecrosis, likely due to both the rare nature of the condition and its often-asymptomatic presentation in comparison to osteonecrosis in greater weight-bearing joints like the hip. Chung et al.^30^ found in a population of 40 sickle cell patients that the prevalence of humeral head osteonecrosis was 3.8%, which was slightly higher than the current study’s prevalence of 2.0% across both chemotherapy and sickle cell etiologies.

Our study found that the prevalence of osteonecrosis lesions within the humeral head among all possible sites of osteonecrosis was 8.5%. Fisher et al.^31^ evaluated a cohort of 77 patients with glucocorticoid-induced osteonecrosis and reported a slightly higher prevalence of 14.3% for osteonecrosis of the humeral head. However, they primarily evaluated adults, with a mean cohort age of 45 years (Range: 17–68).^31^ Additionally, the four studies included in our calculation all involved chemotherapy-induced osteonecrosis. Cruess et al.^32^ also evaluated a cohort of 95 patients with steroid-induced osteonecrosis and found that 18.9% of patients displayed involvement of the humeral head.

In 6 of the 12 studies included in this review, conservative management of humeral head osteonecrosis was explored. Whereas Riccio et al.^23^ and Martin et al.^26^ highlighted that nonsurgical interventions such as physical therapy and activity modification can result in resolution of pain and improved ROM, Wong et al.^28^ provided an example where these types of interventions did not result in pain resolution. Usher et al.^33^ reported that prior to subchondral bone collapse, physical therapy, ROM exercises, and activity modification are available therapies for humeral head osteonecrosis. Kaste et al.^20^ demonstrated the efficacy of intra-articular steroid injections in improving mean CTCAE scores for ROM, pain, and overall impact on ADLs. However, this study is limited in that it was primarily descriptive and did not use hypothesis testing to evaluate whether the improvements were significant.^20^ Wong et al.^28^ showcased an example of the efficacy of intra-articular hyaluronic acid injections; however, no randomized controlled trials or cohort studies have evaluated its efficacy. One important consideration for utilizing intra-articular injections remains the risk of infection in often already immunocompromized patients.^34^ Overall, studies evaluating and comparing the efficacy of different conservative treatments for pediatric humeral head osteonecrosis are lacking.

Three studies in this current study explored surgical management of pediatric humeral head osteonecrosis. Whereas Kaste et al.^20^ demonstrated that mean CTCAE scores improved for pain and impact on ADLs after undergoing CD, Inaba et al.^17^ reported that both shoulders in their cohort that underwent CD did not show resolution of osteonecrosis. Mont et al.^35^ found that in a cohort of 30 shoulders that underwent CD to treat humeral head osteonecrosis, 73% displayed good clinical results over a mean 5.6 years of follow-up. Additionally, every patient with Ficat and Arlet Stage I or II osteonecrosis displayed improvement after CD.^35^ In the current study, Kaste et al.^20^ furthermore reported that resurfacing hemiarthroplasty is an effective treatment for advanced pediatric humeral head osteonecrosis, with eight patients improving their mean CTCAE scores in ROM, pain, and impact on ADLs postoperatively. Orfaly et al.^36^ prospectively evaluated the outcomes of 37 adult total shoulder arthroplasties (TSAs) and 28 adult hemiarthroplasties over a mean 4.3 years, and found that mean visual analog scale pain scores improved from 64 to 12 postoperatively. Patients in both groups experienced significant improvement in function, ADLs, and ROM.^36^ Franceschi et al.^5^ conducted a systematic review comparing CD, hemiarthroplasty, and TSA in adults with humeral head osteonecrosis and found that while CD is effective for low-grade osteonecrosis, arthroplasty should be utilized for high grade osteonecrosis. However, comparing the efficacy of surgical interventions for pediatric humeral head osteonecrosis is challenging given the limited literature.

There are several limitations to this study. First, we were restricted by the available evidence on this topic. Given the rarity of humeral head osteonecrosis, especially in the pediatric population, many of the included studies were limited in sample size and did not include rigorous and robust analyses for the included interventions. Second, it is important to note that when comparing the results of the included studies, the patients were not standardized in demographic characteristics, osteonecrosis grade, and treatment protocols. Thus, the data provided are susceptible to selection, indication, and surveillance bias. Third, some of the studies included were case reports or published before the year 2000. While this may limit their quality or relevance, given the rareness of this condition, they were included in the screening process. Fourth, while this current study captures common nonsurgical and surgical interventions, it does not capture the full scope of interventions as well as the full scope of their clinical and radiographic outcomes using standardized, validated instruments.

This systematic review sought to highlight the prevalence and management of humeral head osteonecrosis—a rare condition with notable morbidity—in the pediatric population. The prevalence of humeral head osteonecrosis across eight of the included studies was about 2%. Given how rare this condition is in the pediatric population, coupled with the limited number of published studies on this topic, it is critical to interpret the results of this study in the context of the limitations above. Overall, the goal of this study is to help surgeons better understand the characteristics of this condition and the treatment options available for management in this population for improved patient and family counseling. Future studies should seek to prospectively evaluate conservative and surgical interventions using validated outcome measures to derive more concrete, comparative evidence on the best management strategies for these patients.

## Supplemental Material

sj-docx-1-cho-10.1177_18632521241254708 – Supplemental material for Management of osteonecrosis of the humeral head in the pediatric population: A systematic review Supplemental material, sj-docx-1-cho-10.1177_18632521241254708 for Management of osteonecrosis of the humeral head in the pediatric population: A systematic review by Vineet M Desai, Akbar N Syed, Morgan Batley, Lawrence Wells and Brendan A Williams in Journal of Children’s Orthopaedics

sj-pdf-2-cho-10.1177_18632521241254708 – Supplemental material for Management of osteonecrosis of the humeral head in the pediatric population: A systematic review Supplemental material, sj-pdf-2-cho-10.1177_18632521241254708 for Management of osteonecrosis of the humeral head in the pediatric population: A systematic review by Vineet M Desai, Akbar N Syed, Morgan Batley, Lawrence Wells and Brendan A Williams in Journal of Children’s Orthopaedics
